# A survey on Apicomplexa protozoa in sheep slaughtered for human consumption

**DOI:** 10.1007/s00436-022-07469-9

**Published:** 2022-02-22

**Authors:** Giorgia Dessì, Claudia Tamponi, Cinzia Pasini, Francesca Porcu, Luisa Meloni, Lia Cavallo, Maria Francesca Sini, Stephane Knoll, Antonio Scala, Antonio Varcasia

**Affiliations:** Parasitology, Department of Veterinary Medicine, University of Sassari, Sassari, Italy

**Keywords:** *Toxoplasma gondii*, *Neospora caninum*, *Sarcocystis tenella*, Sheep, Foodborne parasites, Italy

## Abstract

Infections with the Apicomplexa *Toxoplasma gondii*, *Neospora caninum*, and *Sarcocystis* spp. are common causes of reproductive disorders in sheep. However, few epidemiological studies regarding co-infections with these three protozoa are reported in sheep in Italy. For this reason, this study aims to evaluate possible co-infections with *T. gondii*, *N. caninum*, and *Sarcocystis* spp. in sheep slaughtered for human consumption. From April to July 2019, individual blood, brain, heart, and diaphragm samples were collected from 138 sheep after slaughtering. The presence of IgG anti-*Toxoplasma* in serum samples was evaluated through ELISA. DNA of the three protozoa was investigated using specific PCRs. Co-infection with *T. gondii*, *N. caninum* and *Sarcocystis* spp. was found in 66.7% of the examined sheep. Antibodies against *T. gondii* were found in the 36.2% of serum samples. The presence of *T. gondii* DNA was detected in the 67.4%, 77.5%, and 21.7% of the brain, heart, and diaphragm samples, respectively. *Neospora caninum* DNA was found in 72.5% of the examined brain samples. *Sarcocystis* spp. DNA was detected in 92% and 52.2% of the heart and diaphragm samples, respectively. Sequence analysis of the *Sarcocystis* spp. revealed the sole presence of *Sarcocystis tenella*. The present study demonstrates that sheep have a high risk of infection with the three Apicomplexa investigated, suggesting the need to adopt adequate measures to prevent the spread of these parasitic infections considering their clinical and economic impact on ovine production. Furthermore, the possible role sheep play in the zoonotic transmission of toxoplasmosis to humans was highlighted.

## Introduction

The phylum Apicomplexa includes parasites of veterinary and medical significance as well as economic interest (Ortega-Mora et al. 2007; Gajadhar et al. 2015). Three important protozoa within this phylum, *Toxoplasma gondii*, *Neospora caninum*, and *Sarcocystis* spp., negatively impact the reproductive efficiency of farmed ruminants including sheep (Buxton 1998; Ortega-Mora et al. 2007; Lindsay and Dubey 2020). While *N. caninum* is mainly known to cause reproductive failure in cattle, *T. gondii* is recognized to be one of the principal causes of abortion in sheep (González-Warleta et al. 2014; Hecker et al. 2019). Sarcocystosis in small ruminants is associated with foetal infection and abortions to a lesser extent (Buxton 1998; Ortega-Mora et al. 2007).

*Toxoplasma gondii* and *N. caninum* exhibit a similar two-stage asexual life cycle in the intermediate host and a host-specific sexual cycle in the definite host (Ortega-Mora et al. 2007; Lindsay and Dubey 2020). The first, *T. gondii*, is carried by felids (definitive hosts), and its infective stage is capable of infecting virtually all warm-blooded animals, including humans (Innes et al. 2009; Gajadhar et al. 2015). In sheep, clinical toxoplasmosis appears when the infection occurs during early to mid-gestation. In such cases, spreading tachyzoites can cause transplacental infection (exogenous trans-placental transmission) leading to parasitism of placental and foetal tissues followed by foetal death and resorption, abortion, stillbirth, or weakly born lambs often together with a mummified foetus (Buxton 1998; Taylor 2000; Innes et al. 2009; Lindsay and Dubey 2020). For *N. caninum*, canids act as definitive hosts and ruminants (including sheep) as intermediate hosts (Ortega-Mora et al. 2007; Dubey et al. 2017). Like the previous, tachyzoites disseminate to numerous organs, possibly including trans-placentally to the foetus, causing tissue damage (Ortega-Mora et al. 2007; Dubey et al. 2017). Additionally, as in cattle, it is likely that reactivation of dormant tissue cysts during gestation allowing for tachyzoites to spread to the foetal tissues (endogenous trans-placental transmission) represents a major infection route for this parasite in sheep (González-Warleta et al. 2014, 2018; Filho et al. 2017). Contrary to *T. gondii*, ovine neosporosis was previously not considered to impact the reproductive success of flocks significantly (Buxton 1998; Taylor 2000; Hässig et al. 2003). However, recent reports and studies are pointing towards *N. caninum* to be the cause of reproductive dysgenesis in sheep more often than previously thought (West et al. 2006; Masala et al. 2007; Howe et al. 2012; Moreno et al. 2012; González-Warleta et al. 2014; 2018; Hecker et al. 2019). Besides, experimentally induced neosporosis during the first and second thirds of gestation was shown to be 100% fatal for the foetus, leading to foetal resorption or abortion (Arranz-Solís et al. 2015; Dubey et al. 2017). 

The third protozoa of interest, *Sarcocystis* spp., have an obligatory prey-predator life cycle in which prey ingest sporocysts presents in food or water contaminated by the faeces of predators (Taylor 2000; Lindsay and Dubey 2020). Asexual reproduction occurs in the intermediate host and includes multiple generations of merogony with the formation of bradyzoite sarcocysts in the intermediate host’s muscle cells (Buxton 1998; Gajadhar et al. 2015; Lindsay and Dubey 2020). Overall, sheep function as intermediate hosts for six species of *Sarcocystis*: *Sarcocystis tenella*, *Sarcocystis arieticanis*, *Sarcocystis gigantea*, *Sarcocystis medusiformis*, *Sarcocystis microps*, and *Sarcocystis mihoensis* (Hu et al. 2017; Gjerde et al. 2020). Pathogenic species consist of those with canid definite hosts (*S. tenella*, *S. arieticanis*) (Buxton 1998; Ortega-Mora et al. 2007; Dubey et al. 2015a) where primary infection during gestation can lead to foetal death, abortion, or premature lambs (Taylor 2000; Ortega-Mora et al. 2007; Dubey et al. 2015a).

Antibodies against *T. gondii* have been detected in small ruminants worldwide, and based on previously published reviews, it is clear this parasite to be highly prevalent in sheep (Dubey 2009; Stelzer et al. 2019). Similarly, the presence of *N. caninum* in sheep has been documented in most parts of the world including Europe, the Middle East, Asia, Australia, New Zealand, and South America (Dubey et al. 2017). *Sarcocystis* spp. are some of the most common parasites in livestock (Hecker et al. 2018), and *S. tenella*, *S. arieticanis*, and *S. gigantea* seem to have a global distribution (Dubey et al. 2015a; Gjerde et al. 2020). The presence of *S. medusiformis* has only been recorded in New Zealand, Australia, Iran, Jordan, Spain, and Sardinia, Italy (Dubey et al. 2015a; Gjerde et al. 2020), and *S. microps* and *S. mihoensis* are rarely reported (Hu et al. 2017; Gjerde et al. 2020).

In Italy, depending on the region and technique, a *T. gondii* seroprevalence between 28 and 83% has been reported (Fusco et al. 2007; Masala et al. 2007; Natale et al. 2007; Vesco et al. 2007; Zedda et al. 2010; Cenci-Goga et al. 2013; Gazzonis et al. 2015; Bacci et al. 2016). For *N. caninum*, a seroprevalence of 19–46% can be found in the current scientific literature (Tamponi et al. 2015; Gazzonis et al. 2016). Fewer studies on sarcocystosis in Italian sheep have been published. However, current data suggests pathogenic species to be highly common as the presence of these parasites was detected in 78-100% of examined slaughterhouse samples (Pipia et al. 2016; Bacci et al. 2016; Pagano et al. 2020).

Even though current data show *T. gondii*, *N. caninum* and *Sarcocystis* spp. to be widespread in sheep in Italy, epidemiological studies concerning the co-infection of these three protozoa commonly recognized to cause significant economic losses are scarce. Furthermore, the few studies where multiple ovine Apicomplexa are included solely report two of the three parasites are discussed above (Masala et al. 2007; Bacci et al. 2016; Gazzonis et al. 2016). For this reason, this study aims to evaluate possible co-infections with *T. gondii*, *N. caninum*, and *Sarcocystis* spp. in sheep slaughtered for human consumption in Sardinia, where approximately half (over 3 million of sheep) of the entire Italian sheep population is reared (ISTAT 2020), through the use of biomolecular and serological methods.

## Material and methods

### Sample collection

From April to July 2019, individual blood and tissue samples (brain, heart and diaphragm) were collected, at the time of slaughtering, from 138 Sarda sheep, females, aged between 3 and 7 years and semi-extensively reared. The amount of tissue samples was 50 g for the brain and heart, while 5 g was collected from the diaphragm. Samples were collected in abattoirs from 8 different municipalities in Sardinia. Each animal was assigned a unique ID number, and samples were marked accordingly. Samples were transported to the Parasitology Laboratory of the Veterinary Teaching Hospital of the University of Sassari immediately after collection. Upon arrival, blood samples were centrifuged at 2000 rpm for 10 min, and the obtained sera were stored at – 20 °C. Each tissue sample (brain, heart, and diaphragm) was homogenized into small pieces (approximately 1 mm × 1 mm) using an Ultra Turrax® homogenizer (IKA, Staufen, Germany). All devices used were washed several times with sodium hypochlorite solution (2.5%) followed by distillate water to avoid DNA cross-contamination between the samples, as previously described (Santos et al. 2010). After homogenization, an aliquot of 50 mg was stored at – 20 °C for biomolecular examination.

### Biomolecular analysis

DNA was extracted from 50 mg of homogenized tissue (brain, heart, and diaphragm) using a commercial kit (G-spin^TM^ total DNA extraction kit, Korea), according to the manufacturer instructions. Three different polymerase chain reaction (PCR) protocols were applied to detect the DNA of *T. gondii*, *Sarcocystis* spp., and *N. caninum*, respectively. Each PCR reaction was carried out in a final volume of 25 μl containing 10X PCR buffer, 1.5 mm MgCl_2_, 0.2 mM of each deoxynucleotide triphosphate (dNTP), and 0.2 μM of Thermus aquaticus DNA polymerase (Thermo Fisher Scientific, Massachusetts USA). For all *T. gondii* samples (brain, heart, and diaphragm), a nested PCR (nPCR) was performed in order to amplify a 302 bp fragment of the internal transcribed spacer 1 (ITS1) region as previously described (Halová et al. 2013). In detail, the external primers NN1 (5′-CCTTTGAATCCCAAGCAAAACATGAG-3′) and NN2 (5′-CGAGCCAAGACATCCATTGCTGA-3′) and the internal primers ITSfw (5′-GATTTGCATTCAAGAAGCGTGATAGTAT-3′) and ITSrev (5′-AGTTTAGGAAGCAATCTGAAAGCACATC-3′ were used for the first and second PCR reaction, respectively. The thermal cycler conditions were 94 °C for 3 min, 40 cycles of 94 °C for 30 s, 65 °C for 45 s, and 72 °C for 1 min, followed by 5 min at 72 °C for the first PCR reaction and 95 °C for 5 min, 50 cycles of 94°C for 40 s, and 60°C and 72 °C for 1 min followed by 7 min at 72°C for the second PCR reaction. *Neospora caninum* DNA was detected in brain samples through an nPCR amplifying the 224 bp NC5 target region as reported by Yao et al. (2009). Briefly, the external primers were Np6+ (5′-CTCGCAGTCAACCTACGTCTTCT-3’) and Np21+ (5′-CCCAGTGCGTCCAATCCTGTAAC-3′), while the internal primer were Np9 (5′-GTTGCTCTGCTGACGTGTCGTTG-3′) and Np10 (5′ CTCAACACAGAACACTGAACTCTCG 3′); the thermal cycler conditions were the same for the first and second PCR reactions: 94°C for 5 min, 35 cycles of 94 °C per 30 s, 63 °C for 20 s, and 72 °C for 30 s followed by 10 min at 72 °C. Finally, *Sarcocystis* spp. DNA extracted from heart and diaphragm samples was detected through conventional PCR targeting a fragment of the rRNA 18S gene (609bp) according to Hamidinejat et al. (2014). Specifically, the primers used were Sar-F1 (5′ GCACTTGATGAATTCTGG CA 3’) and Sar-F2 5′ CACCACCCATAGAATCAAG 3′), and the thermal cycler conditions consisted in 94 °C for 5 min, 30 cycles of 94 °C for 2 min, 57°C for 30 s, and 72 °C for 2 min, followed by 72°C for 5 min. All PCR reactions were run in a GeneAmp® PCR System 9700 thermal cycler (Applied Biosystem, Foster City, CA, USA). The PCR amplification products were resolved using electrophoresis in 2% agarose gels and visualized by UVIdoc HD2 (UVITEC, Cambridge, UK). PCR-positive samples were purified using nucleospin gel and PCR clean up (Macherey-Nagel GmbH & Co. KG, Düren, Germany) and sent to an external sequencing service (Eurofins Genomics, Ebersberg, Germany) in order to confirm the specificity of the PCR amplifications. The sequences obtained were compared with those found in the National Centre for Biotechnology Information (NCBI) database using Basic Local Alignment Search Tool (BLAST) (http://www.ncbi.nlm.nih.gov/BLAST/).

### Serological survey

Sera were tested for the presence of IgG anti-*Toxoplasma* using a commercial enzyme linked immuno-adsorbent (ELISA) kit (PrioCHECK® Toxoplasma Ab SR, Prionics, Schlieren-Zurich, Switzerland). The kit included ELISA plates coated with cell culture-derived *T. gondii*-tachyzoite antigens, a peroxidase-labelled anti-small ruminant secondary antibody, tetramethyl benzidine (TMB) as a chromogenic substrate, control sera, and buffer solutions. Serum samples were tested at a 1:100 dilution with sample diluent buffer. Optical density (OD) was measured at a wavelength of 450 nm (reference filter 620 nm), and the results were interpreted by calculating, for each sample, a percentage of positivity (PP) relative to the OD of the positive control (PP sample = OD450 nm sample/OD450 nm positive control × 100). A PP value exceeding 20 was considered as positive and below 20 as negative, as suggested by the manufacturer.

### Statistical analysis

The data generated for *T. gondii*, *N. caninum*, and *Sarcocystis* spp. were recorded on a spreadsheet (Microsoft Excel®, Microsoft Corp., Redmond, WA) and subsequently analysed by Chi-square test (χ^2^) (Epi-info 6.04, CDC, Atlanta, GA, USA). Results were considered statistically significant for *P* < 0.05.

## Results

**Fig. 1:**
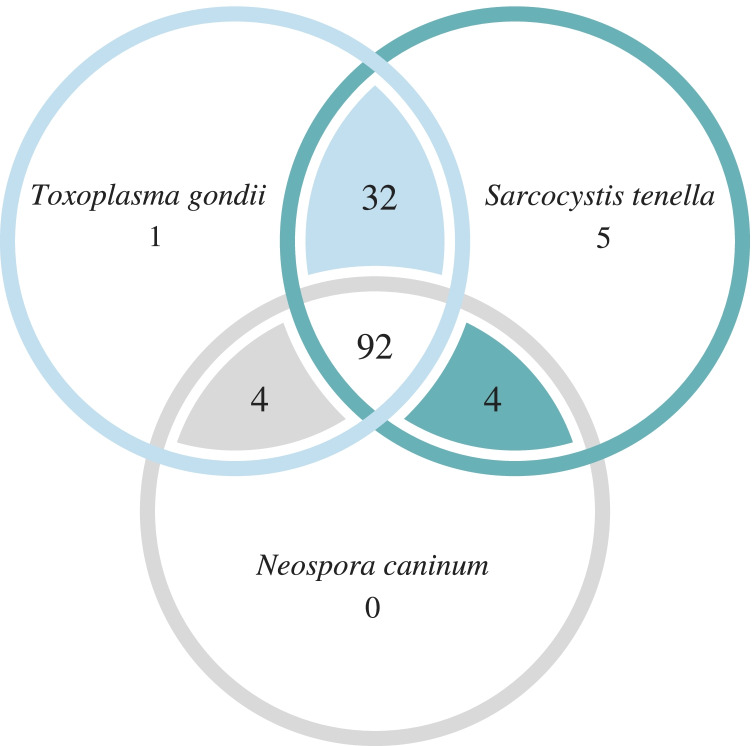
Venn diagram showing the prevalence of *Toxoplasma gondii*, *Neospora caninum* and *Sarcocystis tenella* found in the examined animals and their intersection

All sheep included within this research (100%, 138/138) were found to be positive for at least one of the three targeted protozoa, at least one of matrices and diagnostic techniques used. Co-infection with *T. gondii*, *N. caninum*, and *Sarcocystis* spp. was detected in 66.7% (92/138) of the examined sheep. Details are shown in Fig. 1.

### Biomolecular analysis

The presence of *T. gondii* DNA was detected in the 67.4% (93/138), 77.5% (107/138), and 21.7% (30/138) of the brain, heart, and diaphragm samples, respectively (χ^2^ = 98.75; *P* < 0.001). No statistically significant differences were observed between samples from the heart and brain (χ2 = 3.56; *P* = 0.059), while *T. gondii* prevalence was significantly higher in the heart (χ2 = 85.21; *P* < 0.001) and the brain (χ2 = 58.21; *P* < 0.001) compared to the diaphragm. *Neospora caninum* DNA was found in 72.5% (100/138) of the examined brain samples. The sequence analysis of the NC5 gene identified the isolates as *N. caninum* with a homology of 98.86–100% (accession number LN714488), confirming the PCR amplification results. Finally, ovine heart and diaphragm tissues did not show any macroscopic cysts even though PCR revealed *Sarcocystis* spp. DNA in 92% (127/138) and 52.2% (72/138) of the heart and diaphragm samples, respectively (χ2 = 54.49; *P* < 0.001). Sequencing of the 18s rRNA gene from the *Sarcocystis* spp. isolates showed a homology of 100% with *S. tenella* sequences deposited in GenBank (accession number KP263759).

### Serological survey

Antibodies against *T. gondii* were found in the 36.2% (50/138) of serum samples. In 44 of these 50 seropositive animals, DNA could be detected in the brain, heart, or diaphragm. Thus, 31.9% (44/138) of the examined sheep were positive for *T. gondii* by both methods used, PCR and ELISA.

## Discussion

The present survey highlights the presence of co-infection with *T. gondii*, *N. caninum*, and *Sarcocystis* spp. in sheep slaughtered for human consumption in Sardinia. Additionally, high prevalence rates of these protozoa were recorded in different matrices (serum, brain, heart, and diaphragm tissue samples).

Results herein reported show the widespread presence of *T. gondii* in sheep farms in Sardinia, as recently reported in pigs and cattle on the same island (Pipia et al. 2018; Gazzonis et al. 2020). The seroprevalence of *T. gondii* recorded in the present survey (36.2%) is considerably lower than that reported in Sardinia more than 10 years ago (51.3%) (Natale et al. 2007). Similar results were reported in Portugal (33.6%) and Iran (33.62%) (Lopes et al. 2013; Izadyar et al. 2019).

A higher percentage of positive samples was found using PCR (performed in different matrices) than ELISA (on serum samples). This agrees with other studies where a higher *T. gondii* prevalence was found through PCR compared to serological methods (Rasti et al. 2017; Yousefvand et al. 2021). Such differences most likely result from molecular methods having a higher precision, sensitivity, and specificity than serological methods (Martínez-Flores et al. 2017; Abd El-Razik et al. 2018). Furthermore, through PCR analysis, active *T. gondii* infections can be identified as both living and dead parasites can be detected (Robert-Gangneux and Dardè 2012; Liu et al. 2015). Instead, ELISA test used in the present survey can solely identify chronic infection through the detection of IgG anti-*Toxoplasma* produced by the infected host (Robert-Gangneux and Dardè 2012; Liu et al. 2015).

PCR results showed the prevalence of *T. gondii* to be significantly higher in the heart (77.5%) and brain (67.4%) compared to diaphragm samples (21.7%). Given these results, and in accordance with previous research, authors suggest heart samples to be the best choice for the isolation of *T. gondii* (Dubey et al. 2015b). In addition to skeletal and cardiac muscles, the central nervous system has been proven to be a preference site for tissue cysts (Tenter 2009). However, the distribution of cysts and the parasite burden in different tissues may depend on the *T. gondii* strain, the infective stage (oocysts, tachyzoites and bradyzoites), and the time of infection, increasing in heart and skeletal muscles over time (Dadimoghaddam et al. 2014; Juránková et al. 2014; Swierzy et al. 2014; Yousefvand et al. 2021).

An underestimation of the *T. gondii* prevalence in the diaphragm samples within this research may have occurred because for diaphragm, only 5 g was homogenized, whereas for brain and heart tissue, 50 g of each was homogenized, resulting in a potentially higher chance of including tissue cyst for those latter tissues. Indeed, the distribution of *T. gondii* parasites within tissues is heterogenous, and thus, the parasites could have been present in the unexamined parts of the diaphragm (Robert-Gangneux and Dardè 2012). It is worthy to note the high prevalence of *T. gondii* found in sheep slaughtered for human consumption in this research, revealing a potential risk for consumers, especially pregnant woman and immunodeficient individuals (Weiss and Dubey 2009). Indeed, the consumption of raw or undercooked meat containing *T. gondii* tissue cysts is among the main routes of transmission of this parasite to humans, together with oocyst contaminated water and contact with cat faeces carrying *T. gondii* oocysts (Dubey 2021). Thus, this research allowed to confirm sheep meat as a possible source of toxoplasmosis for humans (Belluco et al. 2016; OIE World Organization for Animal Health 2021). This is especially true considering the popularity of sheep products on the island. Nevertheless, in Sardinia, several projects have been undertaken to improve the global value of sheep meat-based food products, e.g. the production of sheep sausages, ham, and air-dried whole shoulder (Mangia et al. 2006). Although the curing processes applied in their production make these products microbiologically safe for human consumption, these processes have not been validated for the inactivation of *T. gondii* cysts present in meat and require further attention (Herrero et al. 2017; Hill et al. 2018; Fredericks et al. 2020). Finally, given the high presence of *T. gondii* found in the heart and brain of sheep examined within this research, authors recommend at risk people to avoid the consumption of products derived from these sources or better to consume them after having been cooked thoroughly.

A potential risk for the livestock industry is also highlighted here since toxoplasmosis is recognized to be responsible of great economic losses due to foetal death in addition to costs linked to veterinary services (e.g. diagnosis, costs for anti-inflammatory substances to reduce the fever in acute toxoplasmosis, treatment of fertility problems after abortion) (Stelzer et al. 2019; Nayeri et al. 2021).

Similarly, the significance of the high prevalence of *N. caninum* (72.5%) reported here should not be underestimated. Despite that the clinical, epidemiological, and economic importance of *N. caninum* infections in sheep is still unclear, the potential role of this protozoa in ovine reproductive problems has been highlighted by several authors (Howe et al., 2012; González-Warleta et al. 2014; Al-Shaeli et al. 2020). Additionally, *N. caninum* has previously been isolated from ovine abortion samples in Sardinia, emphasizing its causal role in sheep abortions (Masala et al. 2007).

Besides this, the presence of numerous stray and shepherd dogs in Sardinia (and their close contact with sheep) could contribute to the spread of this parasitosis to more sensitive animal species such as cattle in which *N. caninum* is considered the highly prevalent cause of abortion (Al-Shaeli et al. 2020; Varcasia et al. 2020). Luckily there is no evidence of the zoonotic potential of this protozoan even though high frequency of *N. caninum* antibodies has been found in humans, especially immunocompromised patients (Lobato et al. 2006; Duarte et al. 2020).

Most data regarding ovine neosporosis have been obtained through serological essays while those related to the detection of *N. caninum* DNA in naturally infected adult sheep are few (Castañeda-Hernández et al. 2014; Arbabi et al. 2016; Amdouni et al. 2018). Amplification of the NC5 gene is one of the most suitable techniques for the detection of *N. caninum* due to its sensitivity and specificity, allowing for the discrimination between the related apicomplexan parasites examined in the present survey and for the identification of active infections, contrary to serological tests that only indicate parasite exposure (Castañeda-Hernández et al. 2014; Arbabi et al. 2016). Results obtained here confirm the presence of this protozoa in sheep in Sardinia as previously reported in a sero-epidemiological survey where a prevalence of 44.4% and 46.4% was recorded by ELISA in blood and milk, respectively (Tamponi et al. 2015). However, this research reported the prevalence of *Neospora* DNA only in brain samples, chosen as it is the predilection site of this parasite (Dubey 2009). Further studies are needed to assess the presence of *Neospora* DNA in the other tissue samples and anti-*Neospora* antibodies in the serum.

Occurrence of *Sarcocystis* spp. was evaluated in ovine heart and diaphragm samples by PCR and revealed a prevalence of 92% and 52.2%, respectively. The absence of visible macroscopic cysts suggests the presence of microscopic species, and in fact, sequence analysis identified *S. tenella* as the only species involved. This finding is in accordance with a previous survey on sheep sarcocystosis carried out in Sardinia that reported *S. tenella* with a high prevalence (95.5%) (Pipia et al. 2016). These microscopic cysts producing species are considered the most pathogenic in sheep, responsible for fever, loss of appetite, and anaemia (Dubey 1988; Bacci et al. 2016). Furthermore, *S. tenella* can cause abortion or premature birth of offspring in pregnant sheep (Bacci et al. 2016). Other, less pathogenic species, *S. gigantea* and *S. medusiformis*, were not observed in heart and diaphragm tissues of all the examined animals. These macroscopic *Sarcocystis* transmitted by felids can be found in various tissues and organs depending on the species: *S. gigantea* is mainly detected in the oesophagus, larynx, and tongue, while *S. medusiformis* cysts are found in the diaphragm, abdominal muscles, and the carcass (Dong et al. 2018). A previous study carried out in Sardinia showed the oesophagus and abdominal muscles of sheep to be the most affected by these macroscopic species of *Sarcocystis*, while no macroscopic cysts were found in the diaphragm and heart, in agreement with our results (Pipia et al. 2016). Furthermore, *S. tenella* and *S. arieticanis* were reported as more prevalent in comparison to *S. gigantea* and *S. medusiformis* in China, Brazil, and Iraq (Dong et al. 2018; Minuzzi et al. 2019; Abdullah 2021).

The detection of canid transmitted sarcocystosis in the examined sheep underlines the significant role of dogs in the spread of *S. tenella* among others (such as *N. caninum*) on the island. In Sardinia, sheep flocks have a great chance of coming into contact with dog faeces considering the extensive farming practices applied, the high presence of shepherd dogs, and the defecation behaviour of dogs in general (which increase the environmental spread of various parasites, including *S. tenella*, and the risk of transmitting infection to sheep during grazing) (Smith et al. 2014; Varcasia et al. 2020). Likewise, the extensive sheep farming applied in Sardinia contributes to the contact risk of sheep with cat faeces and the consequent transmission of parasites such as *S. gigantea* and *S. medusiformis*. However, the cat’s defecation behaviour, consisting of burying its faeces, leads to a lower spreading potential of oocysts and sporocysts, decreasing the risk of infection for sheep, possibly explaining the absence of *Sarcocystis* species transmitted by cats in the sheep examined (Tamponi et al. 2020; El-Morsey et al. 2021). On the other hand, for *T. gondii*, where cats also function as definitive hosts, a high prevalence was found, highlighting the possibility that vertical transmission could play an important role in the transmission of this protozoa in sheep (Minuzzi et al. 2019).

Despite that the species responsible for sarcocystosis in sheep (and *Sarcocystis* spp. in general) are host specific (Dubey et al. 2015a), their implication in the development of toxic effects has been studied experimentally in other animal species. In particular, protein extracted from *S. gigantea* was be found to be toxic in mice and rats (Al-Hyali et al. 2009, 2010) and an antigen obtained from *S. tenella* caused toxic manifestations in rabbits (Mandour 1969). In any case, ovine *Sarcocystis* are believed to be non-zoonotic (Dubey et al. 2015a).

In conclusion, the present study demonstrates that sheep have a high risk of infection with the three Apicomplexa investigated (*T. gondii*, *N. caninum*, and *Sarcocystis* spp.) and co-infections are frequent. Our results suggest the brain and heart to be suitable matrices for the molecular detection of the investigated protozoa (*T. gondii*: brain and heart, *N. caninum*: brain, *S. tenella*: heart). Overall, any ovine meat for human consumption should be cooked or prepared adequately in order to inactivate infective parasite stages. Finally, adequate control programs and sanitary measures (e.g. promotion of appropriate disposal of ovine placentas and carcasses, avoiding unsupervised home slaughtering, limiting the access of dogs and cats to livestock) should be adopted in order to prevent the spread of these parasitic infections considering their clinical and economic impact on ovine production and the possible role sheep play in the zoonotic transmission of toxoplasmosis to humans.

## Data Availability

All relevant data generated during this study are included in the article.

## References

[CR1] Abd El-Razik KA, Barakat AMA, Hussein HA, Younes AM, Elfadaly HA, Eldebaky HA, Soliman YA (2018). Seroprevalence, isolation, molecular detection and genetic diversity of *Toxoplasma gondii* from small ruminants in Egypt. J Parasit Dis.

[CR2] Abdullah SH (2021) Investigation of *Sarcocystis* spp. in slaughtered cattle and sheep by peptic digestion and histological examination in Sulaimani Province, Iraq. Vet World 14(2):468-474 10.14202/vetworld.2021.468-474 10.14202/vetworld.2021.468-474 PMC7994137 33776313

[CR3] Al-Hyali NS, Khalil LY, Aljawady MA (2009). Sarcotoxin effect on leukocytic finding and phagocytic activity in mice. J Anim Vet Adv.

[CR4] Al-Hyali NS, Aljawady MA, Mohammad-Fakhri MA (2010). The influence of some physio-chemical properties of sarcotoxin in rats. J Anim Vet Adv.

[CR5] Al-Shaeli SJJ, Ethaeb AM, Gharban HAJ (2020) Molecular and histopathological identification of ovine neosporosis (*Neospora caninum*) in aborted ewes in Iraq. Vet World 13(3):597–603 10.14202/vetworld.2020.597-603 10.14202/vetworld.2020.597-603 PMC7183467 32367970

[CR6] Amdouni Y, Rjeibi MR, Awadi S, Rekik M, Gharbi M (2018). First detection and molecular identification of *Neospora caninum* from naturally infected cattle and sheep in North Africa. Transbound Emerg Dis.

[CR7] Arbabi M, Abdoli A, Dalimi A, Pirestani M (2016). Identification of latent neosporosis in sheep in Tehran, Iran by polymerase chain reaction using primers specific for the Nc-5 gene. Onderstepoort J Vet Res.

[CR8] Arranz-Solís D, Benavides J, Regidor-Cerrillo J, Fuertes M, Ferre I, Ferreras M, Collantes-Fernández E, Hemphill A, Pérez V, Ortega-Mora LM (2015). Influence of the gestational stage on the clinical course, lesional development and parasite distribution in experimental ovine neosporosis. Vet Res.

[CR9] Bacci C, Vismarra A, Passeri B, Sciarrone F, Mangia C, Genchi M, Fabbi M, Vicari N, Bruini I, Brindani F, Kramer L (2016). Detection of *Toxoplasma gondii* and *Sarcocystis tenella* in indigenous Cornigliese sheep in Italy using serological and molecular methods. Small Ruminant Res.

[CR10] Buxton D (1998) Protozoan infections (*Toxoplasma gondii*, *Neospora caninum* and *Sarcocystis* spp.) in sheep and goats: recent advances. Vet Res 29(3-4):289–310 9689743

[CR11] Belluco S, Mancin M, Conficoni D, Simonato G, Pietrobelli M, Ricci A (2016). Investigating the determinants of *Toxoplasma gondii* prevalence in meat: a systematic review and meta-regression. PloS One.

[CR12] Castañeda-Hernández A, Cruz-Vázquez C, Medina-Esparza L (2014). *Neospora caninum*: seroprevalence and DNA detection in blood of sheep from Aguascalientes, Mexico. Small Ruminant Res.

[CR13] Cenci-Goga BT, Ciampelli A, Sechi P, Veronesi F, Moretta I, Cambiotti V, Thompson PN (2013). Seroprevalence and risk factors for *Toxoplasma gondii* in sheep in Grosseto district, Tuscany, Italy. BMC Vet Res.

[CR14] Dadimoghaddam Y, Daryani A, Sharif M, Ahmadpour E, Hossienikhah Z (2014). Tissue tropism and parasite burden of *Toxoplasma gondii* RH strain in experimentally infected mice. Asian Pac J Trop Med.

[CR15] Dong H, Su R, Wang Y, Tong Z, Zhang L, Yang Y, Hu J (2018). *Sarcocystis* species in wild and domestic sheep (*Ovis ammon* and *Ovis aries*) from China. BMC Vet Res.

[CR16] Duarte PO, Oshiro LM, Zimmermann NP, Csordas BG, Dourado DM, Barros JC, Andreotti R (2020). Serological and molecular detection of *Neospora caninum* and *Toxoplasma gondii* in human umbilical cord blood and placental tissue samples. Sci Rep.

[CR17] Dubey JP (1988). Lesions in sheep inoculated with *Sarcocystis tenella* sporocysts from canine feces. Vet Parasitol.

[CR18] Dubey JP (2009). History of the discovery of the life cycle of *Toxoplasma gondii*. Int J Parasitol.

[CR19] Dubey JP (2021). Outbreaks of clinical toxoplasmosis in humans: five decades of personal experience, perspectives and lessons learned. Parasit Vectors.

[CR20] Dubey JP, Calero-Bernal R, Rosenthal BM, Speer CA., Fayer R (2015a) Sarcocystosis of animals and humans. Second Edition, CRC Press, Boca Raton. 10.1201/b19184

[CR21] Dubey JP, Lehmann T, Lautner F, Kwok OC, Gamble HR (2015). Toxoplasmosis in sentinel chickens (*Gallus domesticus*) in New England farms: seroconversion, distribution of tissue cysts in brain, heart, and skeletal muscle by bioassay in mice and cats. Vet Parasitol.

[CR22] Dubey JP, Hemphill, A, Calero-Bernal R, Schares G (2017) Neosporosis in animals. First edition, CRC Press, Boca Raton10.1201/9781315152561

[CR23] El-Morsey A, Abdo W, Zaid AAA, Sorour SSG (2021). Morphologic and molecular identification of three macroscopic *Sarcocystis* species infecting domestic sheep (*Ovis aries*) and cattle (*Bos taurus*) in Egypt. Parasitol Res.

[CR24] Filho PCGA, Oliveira JMB, Andrade MR, Silva JG, Kim PCP, Almeida JC, Porto WJN, Mota RA (2017). Incidence and vertical transmission rate of *Neospora caninum* in sheep. Comp Immunol Microbiol Infect Dis.

[CR25] Fredericks J, Hawkins-Cooper DS, Hill DE, Luchansky JB, Porto-Fett A, Shoyer BA, Fournet VM, Urban JF, Dubey JP (2020). Inactivation of *Toxoplasma gondii* bradyzoites after salt exposure during preparation of dry-cured hams. J Food Prot.

[CR26] Fusco G, Rinaldi L, Guarino A, Proroga YTR, Pesce A, Cringoli G (2007). *Toxoplasma gondii* in sheep from the Campania region (Italy). Vet Parasitol.

[CR27] Gajadhar AA, Lalonde LF, Al-Adhami B, Singh BB, Lobanov V (2015) Foodborne apicomplexan protozoa: Coccidia. In Gajadhar A (Ed.), Foodborne Parasites: in The Food Supply Web, Elsevier, pp. 101–147.10.1016/B978-1-78242-332-4.00006-0

[CR28] Gazzonis AL, Garcia GA, Zanzani SA, Mora LMO, Invernizzi A, Manfredi MT (2016). *Neospora caninum* infection in sheep and goats from north-eastern Italy and associated risk factors. Small Ruminant Res.

[CR29] Gazzonis AL, Marino AMF, Garippa G, Rossi L, Mignone W, Dini V, Giunta RP, Luini M, Villa L, Zanzani SA, Manfredi MT (2020). *Toxoplasma gondii* seroprevalence in beef cattle raised in Italy: a multicenter study. Parasitol Res.

[CR30] Gazzonis AL, Veronesi F, Di Cerbo AR, Zanzani SA, Molineri G, Moretta I, Moretti A, Piergili Fioretti D, Invernizzi A, Manfredi MT (2015). *Toxoplasma gondii* in small ruminants in Northern Italy - prevalence and risk factors. Ann Agric Environ Med.

[CR31] Gjerde B, De la Fuente C, Alunda JM, Luzón M (2020). Molecular characterisation of five *Sarcocystis* species in domestic sheep (*Ovis aries*) from Spain. Parasitol Res.

[CR32] González-Warleta M, Castro-Hermida JA, Regidor-Cerrillo J, Benavides J, Álvarez-García G, Fuertes M, Ortega-Mora LM, Mezo M (2014). *Neospora caninum* infection as a cause of reproductive failure in a sheep flock. Vet Res.

[CR33] González-Warleta M, Castro-Hermida JA, Calvo C, Pérez V, Gutiérrez-Expósito D, Regidor-Cerrillo J, Ortega-Mora LM, Mezo M (2018). Endogenous transplacental transmission of *Neospora caninum* during successive pregnancies across three generations of naturally infected sheep. Vet Res.

[CR34] Halová D, Mulcahy G, Rafter P, Turčeková L, Grant T, de Waal T (2013). *Toxoplasma gondii* in Ireland: seroprevalence and novel molecular detection method in sheep, pigs, deer and chickens. Zoonoses Public Health..

[CR35] Hamidinejat H, Moetamedi H, Alborzi A, Hatami A (2014). Molecular detection of *Sarcocystis* species in slaughtered sheep by PCR-RFLP from south-western of Iran. J Parasit Dis.

[CR36] Hässig M, Sager H, Reitt K, Ziegler D, Strabel D, Gottstein B (2003). *Neospora caninum* in sheep: a herd case report. Vet Parasitol.

[CR37] Hecker YP., Masson FM., Armendano JI, Cora J, Olivares CF, Gual I, Pardini L, Moore DP, Moré G, Cantón GJ (2018) Evaluation of frequency of antibodies against *Toxoplasma gondii*, *Neospora caninum* and *Sarcocystis* spp. and transmission routes in sheep from Humid Pampa, Argentina. Acta Parasitol 63(2):416–421. 10.1515/ap-2018-0048 10.1515/ap-2018-0048 29654669

[CR38] Hecker YP, Morrell EL, Fiorentino MA, Gual I, Rivera E, Fiorani F, Dorsch MA, Gos ML, Pardini LL, Scioli MV, Magariños S, Paolicchi FA, Cantón GJ, Moore DP (2019). Ovine abortion by *Neospora caninum*: first case reported in Argentina. Acta Parasitol.

[CR39] Herrero L, Gracia MJ, Pérez-Arquillué C, Lázaro R, Herrera A, Bayarri S (2017). *Toxoplasma* gondii in raw and dry-cured ham: the influence of the curing process. Food Microbiol.

[CR40] Hill DE, Luchansky J, Porto-Fett A, Gamble HR, Fournet VM, Hawkins-Cooper DS, Urban JF, Gajadhar AA, Holley R, Juneja VK, Dubey JP (2018). Rapid inactivation of *Toxoplasma gondii* bradyzoites during formulation of dry cured ready-to-eat pork sausage. Food Waterborne Parasitol.

[CR41] Howe L, Collett MG, Pattison RS, Marshall J, West DM, Pomroy WE (2012). Potential involvement of *Neospora caninum* in naturally occurring ovine abortions in New Zealand. Vet Parasitol.

[CR42] Hu JJ, Huang S, Wen T, Esch GW, Liang Y, Li HL (2017) *Sarcocystis* spp. in domestic sheep in Kunming City, China: prevalence, morphology, and molecular characteristics. Parasite 24:30. 10.1051/parasite/2017025 10.1051/parasite/2017025 PMC5539603 28766501

[CR43] Innes EA, Bartley PM, Buxton D, Katzer F (2009). Ovine toxoplasmosis. Parasitology.

[CR44] ISTAT Istituto Nazionale di Statistica (2020). http://dati.istat.it/Index.aspx?DataSetCode=DCSP_CONSISTENZE Accessed 13 December 2021

[CR45] Izadyar N, Abd Nikfarjam B, Esmaeili Rastaghi AR, Alizadeh SA, Heydarian P, Saraei M (2019). A serologic study on *Toxoplasma gondii* infection in slaughtered sheep and goats in Qazvin Province, Iran. Trop Anim Health Prod.

[CR46] Juránková J, Basso W, Neumayerová H, Baláž V, Jánová E, Sidler X, Deplazes P, Koudela B (2014). Brain is the predilection site of *Toxoplasma gondii* in experimentally inoculated pigs as revealed by magnetic capture and real-time PCR. Food Microbiol.

[CR47] Lindsay DS, Dubey JP (2020). Neosporosis, toxoplasmosis, and sarcocystosis in ruminants: an update. Vet Clin North Am Food Anim Pract.

[CR48] Liu Q, Wang ZD, Huang SY, Zhu XQ (2015). Diagnosis of toxoplasmosis and typing of *Toxoplasma gondii*. Parasite Vector.

[CR49] Lobato J, Silva DA, Mineo TW, Amaral JD, Segundo GR, Costa-Cruz JM, Ferreira MS, Borges AS, Mineo JR (2006). Detection of immunoglobulin G antibodies to *Neospora caninum* in humans: high seropositivity rates in patients who are infected by human immunodeficiency virus or have neurological disorders. Clin Vaccine Immunol.

[CR50] Lopes AP, Dubey JP, Neto F, Rodrigues A, Martins T, Rodrigues M, Cardoso L (2013). Seroprevalence of *Toxoplasma gondii* infection in cattle, sheep, goats and pigs from the North of Portugal for human consumption. Vet Parasitol.

[CR51] Mandour AM (1969). Studies on the toxicity of *Sarcocystis*. J Med Microbiol.

[CR52] Mangia NP, Murgia MA, Garau G, Merella R, Deiana P (2006) Sardinian fermented sheep sausage: microbial biodiversity resource for quality improvement. In: Olaizola A (ed.), Boutonnet JP(ed.), Bernués A (ed.) Mediterranean livestock production: uncertainties and opportunities. CIHEAM/CITA/CIT, Zaragoza p. 273–277

[CR53] Martínez-Flores WA, Palma-García JM, Caballero-Ortega H, Del Viento-Camacho A, López-Escamilla E, Martínez-Hernández F, Maravilla P (2017). Genotyping *Toxoplasma gondii* with the B1 gene in naturally infected sheep from an endemic region in the pacific coast of Mexico. Vector Borne Zoonotic Dis.

[CR54] Masala G, Porcu R, Daga C, Denti S, Canu G, Patta C, Tola S (2007). Detection of pathogens in ovine and caprine abortion samples from Sardinia, Italy, by PCR. J Vet Diagn Invest.

[CR55] Minuzzi CE, Cezar AS, Bräunig P, Portella LP, Rodrigues FS, Sangioni LA, Vogel FSF (2019) Occurrence of *Sarcocystis gigantea* macrocysts and high frequency of *S. tenella* microcysts in sheep from southern Brazil. Vet Parasitol Reg Stud Rep 15:100256. 10.1016/j.vprsr.2018.12.002 10.1016/j.vprsr.2018.12.002 30929933

[CR56] Moreno B, Collantes-Fernández E, Villa A, Navarro A, Regidor-Cerrillo J, Ortega-Mora LM (2012). Occurrence of *Neospora caninum* and *Toxoplasma gondii* infections in ovine and caprine abortions. Vet Parasitol.

[CR57] Natale A, Porqueddu M, Capelli G, Mocci G, Marras A, Sanna Coccone GN, Garippa G, Scala A (2007). Sero-epidemiological update on sheep toxoplasmosis in Sardinia, Italy. Parassitologia.

[CR58] Nayeri T, Sarvi S, Moosazadeh M, Daryani A (2021). Global prevalence of *Toxoplasma gondii* infection in the aborted fetuses and ruminants that had an abortion: a systematic review and meta-analysis. Vet Parasitol.

[CR59] OIE World Organization for Animal Health (2021) Manual of diagnostic tests and vaccines for terrestrial animals 2021. https://www.oie.int/en/what-we-do/standards/codes-and-manuals/terrestrial-manual-online-access/. Accessed 13 December 2021

[CR60] Ortega-Mora L, Gottstein B, Conraths FJ, Buxton D (2007) Protozoal abortion in farm ruminants: guidelines for diagnosis and control. CAB Int. 10.1079/9781845932114.0000

[CR61] Pagano TB, Prisco F, De Biase D, Piegari G, Maurelli MP, Rinaldi L, Cringoli G, Papparella S, Paciello O (2020). Muscular sarcocystosis in sheep associated with lymphoplasmacytic myositis and expression of major histocompatibility complex class I and II. Vet Pathol.

[CR62] Pipia AP, Varcasia A, Dessì G, Panzalis R, Gai C, Nonnis F, Veronesi F, Tamponi C, Scala A (2018). Seroepidemiological and biomolecular survey on *Toxoplasma gondii* infection on organic pig farms. Parasitol Res.

[CR63] Pipia AP, Varcasia A, Zidda A, Dessì G, Panzalis R, Tamponi C, Marrosu R, Tosciri G, Sanna G, Dore F, Chiesa F, Scala A (2016). Cross-sectional investigation on sheep sarcosporidiosis in Sardinia, Italy. Vet Parasitol Reg Stud Rep.

[CR64] Rasti S, Marandi N, Abdoli A, Delavari M, Mousavi SGA (2017). Serological and molecular detection of *Toxoplasma gondii* in sheep and goats in Kashan, Central Iran. J Food Saf.

[CR65] Robert-Gangneux F, Dardè ML (2012). Epidemiology of and diagnostic strategies for toxoplasmosis. Clin Microbiol Rev.

[CR66] Santos SL, de Souza Costa K, Gondim LQ, da Silva MS, Uzêda RS, Abe-Sandes K, Gondim LF (2010). Investigation of *Neospora caninum*, *Hammondia* sp., and *Toxoplasma gondii* in tissues from slaughtered beef cattle in Bahia, Brazil. Parasitol Res.

[CR67] Smith AF, Semeniuk CA, Kutz SJ, Massolo A (2014). Dog-walking behaviours affect gastrointestinal parasitism in park-attending dogs. Parasit Vectors.

[CR68] Stelzer S, Basso W, Benavides Silván J, Ortega-Mora LM, Maksimov P, Gethmann J, Conraths FJ, Schares G (2019). *Toxoplasma gondii* infection and toxoplasmosis in farm animals: risk factors and economic impact. Food Waterborne Parasitol.

[CR69] Swierzy IJ, Muhammad M, Kroll J, Abelmann A, Tenter AM, Lüder CG (2014). *Toxoplasma gondii* within skeletal muscle cells: a critical interplay for food-borne parasite transmission. Int J Parasitol.

[CR70] Tamponi C, Knoll S, Tosciri G, Salis F, Dessì G, Cappai MG, Varcasia A, Scala A (2020). Environmental contamination by dog feces in touristic areas of Italy: parasitological aspects and zoonotic hazards. Am J Trop Med Hyg.

[CR71] Tamponi C, Varcasia A, Pipia AP, Zidda A, Panzalis R, Dore F, Dessì G, Sanna G, Salis F, Bjorkman C, Scala A (2015). ISCOM ELISA in milk as screening for *Neospora caninum* in dairy sheep. Large Anim Rev.

[CR72] Taylor M (2000). Protozoal disease in cattle and sheep. In Pract.

[CR73] Tenter AM (2009). *Toxoplasma gondii* in animals used for human consumption. Mem Inst Oswaldo Cruz.

[CR74] Varcasia A, Dessì G, Lattanzio S, Marongiu D, Cuccuru C, Carta S, Meloni MP, Tamponi C, Scala A (2020). Cystic echinococcosis in the endemic island of Sardinia (Italy): has something changed?. Parasitol Res.

[CR75] Vesco G, Buffolano W, La Chiusa S, Mancuso G, Caracappa S, Chianca A, Villari S, Currò V, Liga F, Petersen E (2007). *Toxoplasma gondii* infections in sheep in Sicily, southern Italy. Vet Parasitol.

[CR76] Weiss LM, Dubey JP (2009). Toxoplasmosis: a history of clinical observations. Int J Parasitol.

[CR77] West DM, Pomroy WE, Collett MG, Hill FI, Ridler AL, Kenyon PR, Pattison RS (2006). A possible role for *Neospora caninum* in ovine abortion in New Zealand. Small Rumin Res.

[CR78] Yao L, Yang N, Liu Q, Wang M, Zhang W, Qian WF, Hu YF, Ding J (2009). Detection of *Neospora caninum* in aborted bovine fetuses and dam blood samples by nested PCR and ELISA and seroprevalence in Beijing and Tianjin, China. Parasitology.

[CR79] Yousefvand A, Mirhosseini SA, Ghorbani M, Mohammadzadeh T, Moghaddam MM, Mohammadyari S (2021). Molecular and serological detection and of *Toxoplasma gondii* in small ruminants of southwest Iran and the potential risks for consumers. J Consum Prot Food Saf.

[CR80] Zedda MT, Rolesu S, Pau S, Rosati I, Ledda S, Satta G, Patta C, Masala G (2010). Epidemiological study of *Toxoplasma gondii* infection in ovine breeding. Zoonoses Public Health.

